# One-leg inactivity induces a reduction in mitochondrial oxidative capacity, intramyocellular lipid accumulation and reduced insulin signalling upon lipid infusion: a human study with unilateral limb suspension

**DOI:** 10.1007/s00125-020-05128-1

**Published:** 2020-03-17

**Authors:** Lena Bilet, Esther Phielix, Tineke van de Weijer, Anne Gemmink, Madeleen Bosma, Esther Moonen-Kornips, Johanna A. Jorgensen, Gert Schaart, Dongyan Zhang, Kenneth Meijer, Maria Hopman, Matthijs K. C. Hesselink, D. Margriet Ouwens, Gerald I. Shulman, Vera B. Schrauwen-Hinderling, Patrick Schrauwen

**Affiliations:** 1 NUTRIM, School of Nutrition and Translational Research in Metabolism, Maastricht University Medical Center, Maastricht, the Netherlands; 2 Department of Nutrition and Movement Sciences, Maastricht University Medical Center, P.O. Box 616, 6200 MD Maastricht, the Netherlands; 3 Department of Radiology, Maastricht University Medical Center, Maastricht, the Netherlands; 4 Department of Internal Medicine, Yale University School of Medicine, New Haven, CT USA; 5 Department of Physiology, Radbound University Nijmegen Medical Center, Nijmegen, the Netherlands; 6 Institute of Clinical Biochemistry and Pathobiochemistry, German Diabetes Center, Düsseldorf, Germany; 7 Departments of Cellular & Molecular Physiology, Yale University School of Medicine, New Haven, CT USA

**Keywords:** Fat oxidation, Insulin resistance, Intramyocellular lipid content, Mitochondrial function, Mitochondrial oxidative capacity, Physical inactivity, Unilateral lower-limb suspension

## Abstract

**Aims/hypothesis:**

Physical inactivity, low mitochondrial function, increased intramyocellular lipid (IMCL) deposition and reduced insulin sensitivity are common denominators of chronic metabolic disorders, like obesity and type 2 diabetes. Yet, whether low mitochondrial function predisposes to insulin resistance in humans is still unknown.

**Methods:**

Here we investigated, in an intervention study, whether muscle with low mitochondrial oxidative capacity, induced by one-legged physical inactivity, would feature stronger signs of lipid-induced insulin resistance. To this end, ten male participants (age 22.4 ± 4.2 years, BMI 21.3 ± 2.0 kg/m^2^) underwent a 12 day unilateral lower-limb suspension with the contralateral leg serving as an active internal control.

**Results:**

In vivo, mitochondrial oxidative capacity, assessed by phosphocreatine (PCr)-recovery half-time, was lower in the inactive vs active leg. Ex vivo, palmitate oxidation to ^14^CO_2_ was lower in the suspended leg vs the active leg; however, this did not result in significantly higher [^14^C]palmitate incorporation into triacylglycerol. The reduced mitochondrial function in the suspended leg was, however, paralleled by augmented IMCL content in both musculus tibialis anterior and musculus vastus lateralis, and by increased membrane bound protein kinase C (PKC) θ. Finally, upon lipid infusion, insulin signalling was lower in the suspended vs active leg.

**Conclusions/interpretation:**

Together, these results demonstrate, in a unique human in vivo model, that a low mitochondrial oxidative capacity due to physical inactivity directly impacts IMCL accumulation and PKCθ translocation, resulting in impaired insulin signalling upon lipid infusion. This demonstrates the importance of mitochondrial oxidative capacity and muscle fat accumulation in the development of insulin resistance in humans.

**Trial registration:**

ClinicalTrial.gov NCT01576250.

**Funding:**

PS was supported by a ‘VICI’ Research Grant for innovative research from the Netherlands Organization for Scientific Research (Grant 918.96.618).

**Electronic supplementary material:**

The online version of this article (10.1007/s00125-020-05128-1) contains peer-reviewed but unedited supplementary material, which is available to authorised users.

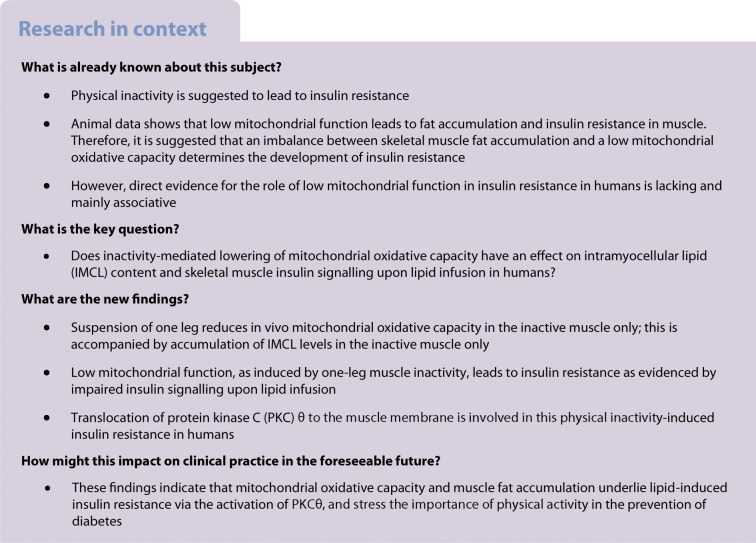



## Introduction

Physical inactivity is a major determinant of the current epidemic of chronic metabolic disorders, like obesity and type 2 diabetes mellitus [1, 2]. Obese individuals may display accumulation of excessive fat in non-adipose tissues, such as liver, heart and skeletal muscle, termed as ectopic fat accumulation [3–5]. Ectopic fat accumulation in skeletal muscle is strongly associated with insulin resistance [6, 7]. Likewise, individuals with type 2 diabetes [8] (as well as first-degree relatives of diabetic individuals), who feature insulin resistance years before the onset of the disease and, therefore, are prone to develop type 2 diabetes [9–11], are characterised by excessive accumulation of fat in skeletal muscle. Increased accumulation of the intramyocellular lipid (IMCL) intermediate diacylglycerol (DAG) and the concomitant activation of protein kinase C (PKC) θ have been reported in the muscle of individuals with type 2 diabetes as an explanation for this lipid-induced insulin resistance [12–15]. However, IMCL content is also increased in highly insulin-sensitive endurance-trained humans (known as the athlete’s paradox) [8], suggesting that IMCL accumulation, per se, is not causal for skeletal-muscle insulin resistance. Studies in lipid-infused rodents have dissociated intramuscular triacylglycerol (TAG) from lipid-induced muscle insulin resistance [16]. In contrast to the well-known high mitochondrial capacity of endurance-trained athletes, several (but not all [17, 18]) studies have found that insulin-resistant individuals with type 2 diabetes are characterised by impaired mitochondrial oxidative capacity [19–22]. These findings suggest that the imbalance between skeletal muscle fat accumulation and a low mitochondrial oxidative capacity determines the development of insulin resistance.

However, studying the direct relationship between physical inactivity, mitochondrial function and insulin resistance in humans is difficult and is mainly derived from cross-sectional studies. A few human experimental studies have applied the model of bed rest to investigate the impact of whole-body altered physical activity on the development of whole-body insulin resistance. To investigate, however, the muscle-specific mechanisms underlying lipid-induced insulin resistance, an unilateral immobilisation study, as presented here, may be helpful. The unilateral lower-limb suspension (ULLS), as originally described by Berg et al. [23], provides a unique model that can be applied in humans to examine the effect of a local reduction in muscle mitochondrial oxidative capacity (as shown previously [24]) on fat accumulation and insulin sensitivity, and allows comparison with an internal unaffected muscle. Here, we aimed to investigate the direct effects of physical inactivity on skeletal muscle mitochondrial oxidative capacity, IMCL accumulation, PKCθ translocation and insulin signalling upon lipid infusion. To this end, we employed the model of ULLS for 12 days in young men, with the contralateral leg serving as an active internal control muscle.

## Methods

### Participants

Ten healthy, lean young men participated in this study. Participant characteristics, as determined before ULLS, are shown in Table 1. The participants included in the study performed exercise maximally two times per week (or less) and none of the volunteers participated in competitive sports. Individuals with unstable body weight (>3 kg change in the 6 months preceding recruitment) were excluded. The institutional medical ethics committee approved the study and all participants gave their informed written consent. The study has been registered with ClinicalTrial.gov (registration no. NCT01576250).

**Table 1:** Participant characteristics (*n* = 10), as measured before ULLS

Characteristic	Mean ± SEM
Age (years)	22.4 ± 4.2
BMI (kg/m^2^)	21.3 ± 2.0
Fat (%)	14.6 ± 3.9
Systolic blood pressure (mmHg)	115 ± 12
Diastolic blood pressure (mmHg)	74 ± 11
Plasma glucose (mmol/l)	4.9 ± 0.3
Plasma TAGs (mmol/l)	1.0 ± 0.4
Plasma NEFA (mmol/l)	0.4 ± 0.2
$\dot{V}{\mathrm{O}}_{2\max }$/kg (ml min^−1^ [kg body weight]^−1^)	48 ± 10

### Study design

**Fig. 1:**
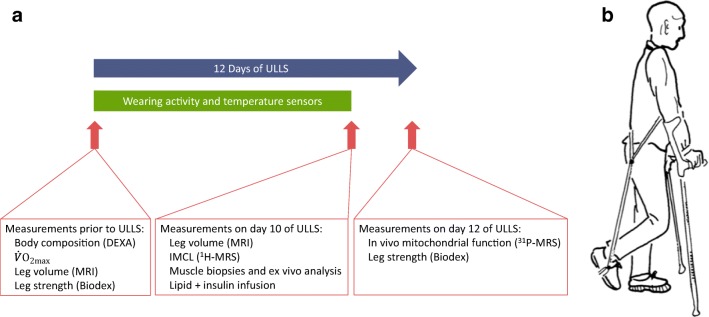
(**a**) Study design and (**b**) ULLS set up. Image in (**b**) from Berg et al. [23], reprinted with permission from the American Physiological Society. DEXA, dual energy x-ray absorptiometry

We examined the effects of one-legged restricted physical activity on skeletal muscle mitochondrial oxidative capacity in vivo, and ex vivo glucose and lipid metabolism, lipid content and insulin signalling. Participants were enrolled in the study between April 2012 until August 2013. Volunteers were subjected to 12 days of ULLS randomised to either their dominant or non-dominant leg. All outcome measurements were performed in the active and the inactivated leg and, where appropriate, in a randomised manner. Outcome parameters were compared within one individual in a paired design. See study design in Fig. 1a.

Before the start of the ULLS intervention, a basal blood sample was obtained after an overnight fast. Furthermore, resting blood pressure, body composition and maximal aerobic capacity was determined in all participants before ULLS.

Lower-limb suspension was performed as previously described [25]. Suspension was achieved by attachment of a sling to a non-rigid ankle brace and to a harness on the upper body. Thus, the suspended leg was unloaded from weight bearing (see Fig. 1b for illustration). The knee was slightly flexed at an angle of 130°, while the hip and the ankle maintained full mobility. The sling was worn during all locomotor activity. Participants were provided with crutches to support daily life mobility. During the night, the sling was taken off. Also, during periods of prolonged sitting and showering the sling was temporarily removed and participants were instructed to minimise activity of the suspended leg during sitting and showering. To monitor compliance and physical activity level, participants: (1) kept a diary of their daily activities; (2) wore temperature sensors on their lower front legs (iButtons; type DS1921H; Maxim Integrated, Dallas, TX, USA), as it is known that a lower physical activity leads to a lower temperature of the leg [26]; and (3) wore leg-specific accelerometers (CIRO Activity Monitors [CAMs]; Maastricht Instruments BV, Maastricht, NL) on both legs at all day times, except for when showering/bathing.

After 9 days of ULLS, participants reported to the laboratory after an overnight fast. Upper leg muscle volume and IMCL content in the muscle tibialis were determined by MRI and proton magnetic resonance spectroscopy (^1^H-MRS), respectively. Thereafter, a basal muscle biopsy was taken from musculus vastus lateralis of both legs, in randomised order, to examine the effect of ULLS on ex vivo lipid metabolism, IMCL content, and incorporation of labelled palmitate into DAG and TAG. Subsequently, a 5 h lipid infusion was initiated to elevate plasma NEFA concentrations to supraphysiological levels previously shown to compromise insulin sensitivity [16]. After 4.5 h of lipid infusion, a short-term hyperinsulinaemic-euglycaemic clamp was performed with simultaneous infusions of insulin and glucose. Insulin was infused at a constant rate and the co-infusion of 20% glucose was adjusted to keep participants in euglycaemia (~5 mmol/l). After 30 min of insulin infusion (and 4.5 h of lipid infusion), a second biopsy was taken from both legs, in a randomised order, to examine the effect of ULLS on markers of insulin sensitivity. On day 12 of ULLS, in vivo mitochondrial oxidative capacity (phosphocreatine [PCr] recovery) and isometric leg strength were measured in both legs using phosphorous magnetic resonance spectroscopy (^31^P-MRS) and Biodex (system 3; Biodex Medical Systems, New York, NY, USA), respectively. During the 2 days separating the clamp procedure and these functional assays, the individuals continued using the sling for ULLS to prevent any carry-over effects of the clamp procedure on subsequent functional assays.

### Maximal performance and body composition

A routine incremental cycling test on a stationary bike to exhaustion was used to assess maximal exercise capacity, as described previously [27] and a dual energy x-ray absorptiometry (DEXA) scan was used to determine body composition. These measurements were done before ULLS.

### Compliance

The activity monitors were attached to the upper part of the suspended and unsuspended limbs, just above the knee, using customised pouches [28]. The CAM contained a triaxial piezoresistive accelerometer and had a sample rate of 25 Hz. MATLAB R2014b (Mathworks, Natick, MA, USA) and algorithms were used to calculate movement intensity [29, 30]. Also skin temperature of the quadriceps and gastrocnemius muscle was monitored using iButtons [31] throughout the whole ULLS period, as a marker of physical activity.

### Muscle volume and strength

As muscle mass is a determinant of insulin sensitivity [32], we aimed to design the intervention such that muscle mass loss would be minimised as a consequence of the suspension by using a relative short duration and a mild level of inactivity. However, as muscle mass can still be changed upon short-term interventions, we examined the muscle volume of the upper leg by serial T2-weighted MRI images, along with measurements of knee flexion and extension strength. Images to assess muscle volume were acquired using a turbo spin echo (TSE) sequence (TSE factor = 21), (Achieva 3Tx; Philips Healthcare, Best, the Netherlands). Twenty contiguous slices of 10 mm thickness were acquired (field-of-view [FOV] = 200× 400× 200 mm; echo time [TE] = 100 ms; repetition time [TR] = 5351 ms; flip angle = 90°, acquired by the body coil; scan duratio*n* = 4 min and 30 s). The most distal slice was positioned on the patella and all slices up to the hip were used for analysis. The magnetic resonance images (in-plane resolution 0.78× 0.78 mm) were segmented into muscle tissue and non-muscle tissue upon automated greyscale-based binning using a home-written script in MATLAB. The muscle area upon segmentation was computed for every single slice and multiplied by slice thickness to compute muscle volume.

Isometric knee extending and flexing strength at five different knee angles (70°, 80°, 90°, 100° and 110°) were measured using a Biodex III dynamometer [33]. Participants were seated upright in a chair and tightly fixed using straps. Subsequently, the knee was brought into position and participants were asked to conduct maximal knee extension (3 s) followed by maximal knee flexion (3 s), while the force traces were recorded on a computer. This procedure was repeated once for each evaluated joint angle. Participants were allowed 2 min of rest between contractions.

### Lipid infusion plus clamp

In the morning after 9 days of limb suspension, participants reported to the laboratory after an overnight fast. After taking basal muscle biopsies (musculus vastus lateralis) from both legs in randomised order, a Teflon cannula was inserted into an antecubital vein for the heparinised (0.2 U kg^−1^ min^−1^) infusion of long-chain TAGs (1.35 ml/min intralipid; Intralipid, Fresenius-Kabi, the Netherlands) for 5 h. Blood was sampled from a second cannula, inserted retrogradely into a superficial dorsal hand vein, for later analysis of plasma glucose and NEFA. This venous blood was arterialised by placing the hand into a hot box that emits warm air (~65°C). After 4.5 h, a simultaneous infusion of insulin (40 mU m^−2^ min^−1^; Novorapid; Novo Nordisk, Copenhagen, Denmark) and glucose was started. During a 30 min hyperinsulinaemic-euglycaemic clamp, plasma glucose levels were clamped at ~5 mmol/l by variable co-infusion of 20% glucose. Thirty min after the start of the insulin infusion, a second muscle biopsy was taken from the vastus lateralis of both legs, in a randomised order.

### Magnetic resonance spectroscopy measurements

In vivo mitochondrial oxidative capacity was determined by ^31^P-MRS in the afternoon after 12 days of ULLS, as previously described [22]. Briefly, a knee-extension protocol was performed on a custom-built magnetic resonance-compatible ergometer with a pulley system in a 3 T whole-body MRI scanner (Achieva 3Tx; Philips Healthcare) for 5 min, with weight corresponding to 50–60% of the participant’s pre-determined maximal knee-extension capacity. A transmit/receive surface coil (5 cm diameter) was positioned on the vastus lateralis muscle and a time series of ^31^P-MRS spectra (free induction decays) were acquired with a repetition time of 4 s. Post-exercise PCr kinetics was computed as previously described [22].

In vivo IMCL content in musculus tibialis was assessed by ^1^H-MRS on a 3.0 T whole-body magnetic resonance system (Achieva 3Tx; Philips Healthcare) as described previously [34]. T2-weighted images were acquired for accurate positioning of the voxel 12× 12× 12 mm^3^ in the tibialis anterior muscle. Point resolved spectroscopy (PRESS) was used with the following acquisition parameters: TR = 2000 ms; TE = 38 ms; number of signal averages (NSA) = 128; sample points = 2048. Water suppression was performed using a selective excitation pulse followed by dephasing gradients. A second non-water suppressed spectrum was acquired subsequently from the same volume (NSA = 8), which enabled us to use the water signal as an internal reference.

The ^1^H-MRS spectra obtained were analysed and fitted in the time domain by using the non-linear least-squares Advanced Method for Accurate, Robust, and Efficient Spectral (AMARES) algorithm [35, 36], in the java-based magnetic resonance user interface (jMRUI) software package [37] as described earlier [34]. IMCL is given as percentage of the CH_2_ peak compared with the water resonance and corrected for T1 and T2 relaxation time. IMCL could not be measured in one participant due to technical failure.

### Muscle biopsies and analysis

Muscle biopsies were taken from the musculus vastus lateralis of both legs after 9 days of ULLS, under local anaesthesia (2% lidocaine; Fresenius-Kabi, Bad Homburg vor der Höhe, Germany), according to the Bergstrom technique [38], and instantaneously processed for ex vivo ^14^C oxidation assays. The remainder of the muscle tissue was stored at −80°C for future analysis of IMCL content by histology, and for markers of insulin action.

[^14^C]palmitate and glucose oxidation assays were performed as previously described [39, 40]. Briefly, palmitate oxidation was determined by measuring production of ^14^CO_2_ and acid-soluble metabolites (ASMs) in skeletal-muscle homogenates containing 250 mmol/l sucrose, 10 mmol/l Tris-HCl, 1 mmol/l EDTA and 2 mmol/l ATP. Reactions were initiated with 0.2 mmol/l palmitate and 0.0175 mmol/l [1-^14^C]palmitate, and terminated with 70% perchloric acid. CO_2_ was trapped in 1 mol/l NaOH. Lipid incorporation was measured by thin-layer chromatography. Radioactivity in bands corresponding to TAG and DAG were quantified by liquid scintillation, as described previously [41]. Glucose oxidation to CO_2_ was determined by replacing [1-^14^C]palmitate with [U-^14^C]glucose.

#### IMCL

IMCL was assessed in muscle cross-sections using a modified Oil Red O staining for fluorescence microscopy [42]. Images were analysed with ImageJ (type 1.51 J8 for windows; NIH, Bethesda, Maryland, USA). Freezing artefacts in sections of four participants prevented valid histological quantification of the IMCL content and these participants were, therefore, excluded from the analysis.

#### Insulin signalling

Muscle biopsies were prepared for analysis of the expression and phosphorylation of components of the insulin signalling pathway by western blotting, as described previously [43]. The blots were incubated overnight at 4°C with antibodies recognising the insulin receptor β-subunit (Santa Cruz Biotechnology, Santa Cruz, CA, USA) and pyruvate dehydrogenase kinase 4 (PDK4) (Abcam, Cambridge, UK), and phosphorylated IRS1^Ser1101^, Akt^Thr308^, Akt^Ser473^, glycogen synthase kinase 3β (GSK3β^Ser9^), glycogen synthase (GS^Ser641^), Forkhead box O (FOXO1^Thr24^/FOXO3a^Thr32^), AMP-activated protein kinase (AMPK^Thr172^) and acetyl-CoA carboxylase (ACC^Ser79^) (all from Cell Signaling Technology, Beverly, MA, USA). For an overview of all antibodies, please see electronic supplementary material (ESM) Table 1. Antibodies were diluted in 5% (wt/vol.) BSA dissolved in Tris-buffered saline and 0.1% Tween-20. Following incubation, the blots were washed with Tris-buffered saline and 0.1% Tween-20. Bound antibodies were detected by incubation with the appropriate secondary horseradish peroxidase (HRP)-conjugated antibodies (Promega, Mannheim, Germany), and visualised by enhanced chemiluminescence on a VersaDoc 4000 MP (Bio-Rad Laboratories, Irvine, CA, USA) workstation. Signals were quantified using ImageLab software (version 5.2.1; Bio-Rad), and normalised for abundance of β-actin and glyceraldehyde 3-phosphate dehydrogenase (GAPDH) (blots shown in ESM Fig. 1).

#### PKCθ translocation

Membrane translocation of PKCθ was assessed as described [16]. Due to lack of muscle material, PKCθ translocation could only be measured in *n* = 8 participants. PKCθ translocation was expressed as the ratio of membrane to cytosol bands on the same film.

### Blood sample analysis

Blood samples were collected in EDTA-containing tubes and immediately spun at high speed, frozen in liquid nitrogen and stored at −80°C. Plasma NEFA, TAGs, and glucose were measured with enzymatic assays automated on a Cobas Fara/Mira (NEFA: Wako NEFA C test kit, Wako Chemicals, Neuss, Germany; glucose: hexokinase method, Roche, Basel, Switzerland; TAGs: ABX Pentra CP reagents, Horiba ABX Diagnostics, Montpellier, France).

### Statistics

All data were analysed in a blinded fashion. Data are presented as mean ± SEM. The samples size was determined based on insulin-stimulated skeletal muscle glucose disposal, with the expected effect size and SD based on previous research within our research group [44]. All statistics were performed using SPSS 16.0 for Mac (IBM, New York, NY, USA). For most parameters, differences between the suspended and the active leg were analysed with a two-tailed, paired Student’s *t* test, unless otherwise specified. The non-normally distributed insulin action data were tested for differences using a non-parametric Wilcoxon signed-rank test for related samples. Statistical significance was set at *p* ≤ 0.05.

## Results

**Fig. 2:**
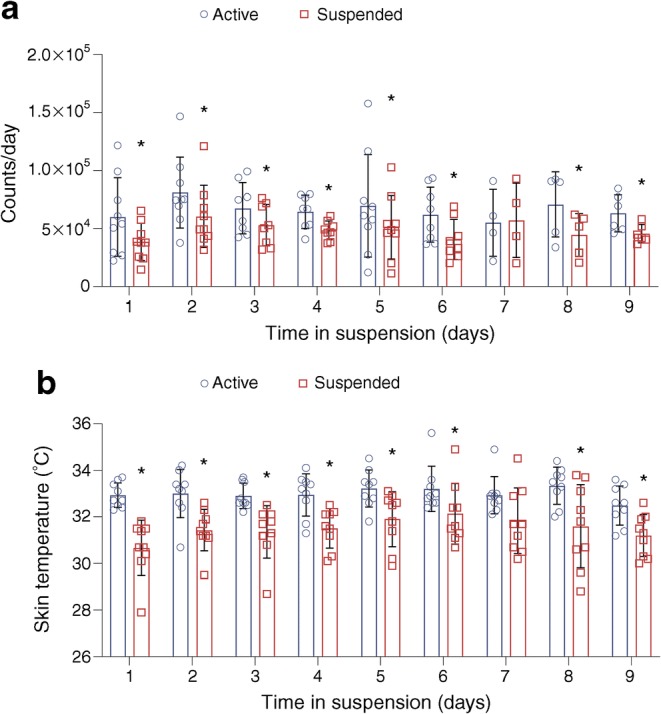
Participant compliance to the intervention as measured with (**a**) activity monitors (accelerometers) (*n* = 4–9) and (**b**) temperature sensors (*n* = 9). *p < 0.05. Data expressed as mean ± SEM

To check for compliance to the suspension protocol, we used both accelerometer data and data on skin temperature; activity counts in the suspended leg were lower in all participants than in the active leg (48,826 ± 7354 counts/day vs 65,969 ± 10,101 counts/day, respectively; *p* < 0.050; Fig. 2a). In line with lower activity, skin temperature was lower in the suspended leg compared with the active leg (lower leg: 31.5 ± 0.03°C vs 33.0 ± 0.02°C; upper leg: 32.2 ± 0.02°C vs 33.0 ± 0.04°C, respectively; *p* < 0.050; Fig. 2b). Jointly, these data indicate that all individuals were very compliant and adhered well to the ULLS protocol.

We were anticipating that upon 10 days of ULLS, mitochondrial oxidative capacity would decrease without large effects on muscle volume and strength. As anticipated, upper leg muscle volume upon ULLS was similar in the active vs the suspended leg (2766 ± 173 cm^3^ in the active leg vs 2794 ± 182 cm^3^ in the suspended leg; *p* = 0.33; data not shown). Furthermore, maximal isometric strength of the knee extensors and flexors was unaffected by ULSS (170 ± 21 Nm for the suspended leg extensor vs 178 ± 20 Nm for the active leg extensor, *p* = 0.601; 95 ± 9 Nm for the suspended leg flexor vs 95 ± 9 Nm for the active leg flexor, *p* = 0.948; data not shown). Data for the knee extension was obtained at an angle of 90° and data for the knee flexion was obtained at an angle of 70°.

**Fig. 3:**
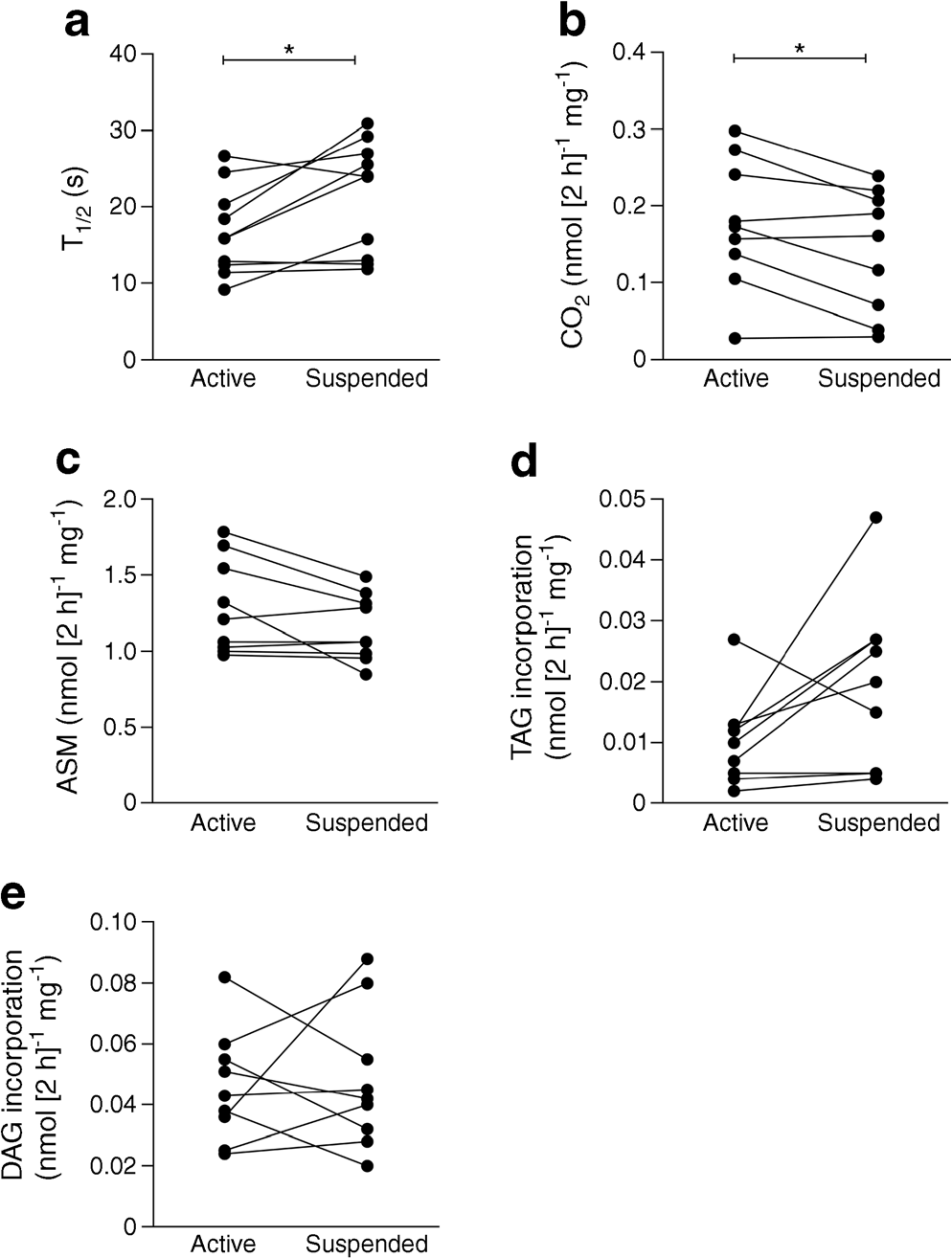
Mitochondrial oxidative capacity and incorporation of labelled palmitate into DAG and TAG in the active vs the suspended leg, post-suspension, in the overnight fasted state. (**a**–**c**) Mitochondrial oxidative capacity expressed as (**a**) PCr-recovery T_1/2_ in vivo (*n* = 10), and (**b**,**c**) ex vivo [^14^C]palmitate oxidation to CO_2_ (**b**; *n* = 9) and ASMs (**c**; *n* = 9). (**d**, **e** (both *n* = 9) [^14^C]palmitate incorporation into TAGs (**d**) and DAGs (**e**). *p < 0.05

Next, we investigated the effect of ULLS on mitochondrial oxidative capacity in vivo by ^31^P-MRS and ex vivo by [^14^C]palmitate oxidation assays. PCr-recovery half-time (T_1/2_) was significantly longer in the suspended leg compared with the active leg, (21.4 ± 2.3 s vs 16.7 ± 1.8 s, respectively; *p* = 0.019; Fig. 3a), demonstrating that mitochondrial oxidative capacity was indeed reduced in skeletal muscle of the inactive leg. Ex vivo palmitate oxidation to ^14^CO_2_ (‘complete oxidation’) was significantly lower in the suspended leg compared with the active leg after 9 days of suspension (0.14 ± 0.03 nmol 2 h^−1^ mg^−1^ vs 0.18 ± 0.03 nmol 2 h^−1^ mg^−1^, respectively; *p* = 0.013; Fig. 3b [*n* = 9]). The level of ASMs (reflecting incomplete oxidation) was non-significantly lower in the suspended leg compared with the active leg (1.15 ± 0.07 nmol [2 h]^−1^ mg^−1^ vs 1.29 ± 0.10 nmol [2 h]^−1^ mg^−1^, respectively; *p* = 0.065; Fig. 3c [*n* = 9]). In addition, incorporation of [^14^C]palmitate into TAGs was non-significantly higher in the suspended leg compared with the active leg (0.019 ± 0.005 nmol [2 h]^−1^ mg^−1^ vs 0.010 ± 0.002 nmol [2 h]^−1^ mg^−1^, respectively; *p* = 0.075; Fig. 3d [*n* = 9]), whereas [^14^C]palmitate incorporation into DAGs was not different between the two legs (0.048 ± 0.008 nmol [2 h]^−1^ mg^−1^ vs 0.046 ± 0.006 nmol [2 h]^−1^ mg^−1^ in the suspended vs the active leg; *p* = 0.822; Fig. 3e). No effects were observed on ex vivo glucose oxidation rates (0.429 ± 0.125 nmol [2 h]^−1^ mg^−1^ in the suspended leg vs 0.567 ± 0.167 nmol [2 h]^−1^ mg^−1^ in the active leg; *p* = 0.461; *n* = 9; data not shown).

**Fig. 4:**
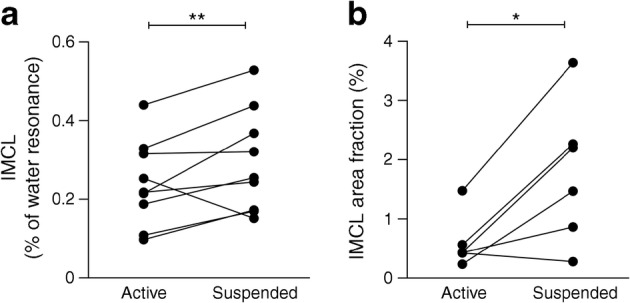
(**a**) In vivo IMCL in the musculus tibialis anterior, measured by ^1^H-MRS, in the active vs the suspended leg, post-suspension, in the overnight fasted state (*n* = 9). (**b**) Ex vivo IMCL in musculus vastus lateralis, measured with Oil Red O staining, in the overnight fasted state (*n* = 6). *p < 0.05, **p < 0.01

We subsequently investigated if the reduction in mitochondrial oxidative capacity in the suspended leg was also paralleled by accumulation of IMCL. These examinations were performed in two different ways: in muscle biopsies taken prior to lipid infusion and with ^1^H-MRS-based measurements of IMCL, both in the fasted state. In vivo IMCL in the musculus tibialis anterior, as measured with ^1^H-MRS, was 23% higher in the suspended leg compared with the active leg (0.31% ± 0.05% vs 0.24% ± 0.04%; *p* = 0.003; Fig. 4a [*n* = 9]). This increase in IMCL upon leg suspension was confirmed in muscle biopsies taken from the musculus vastus lateralis, showing a 53% higher IMCL content in the suspended leg compared with the active leg (lipid area fraction: 1.79% ± 0.48% vs 0.85% ± 0.24%; *p* = 0.021; Fig. 4b [*n* = 6]).

**Fig. 5:**
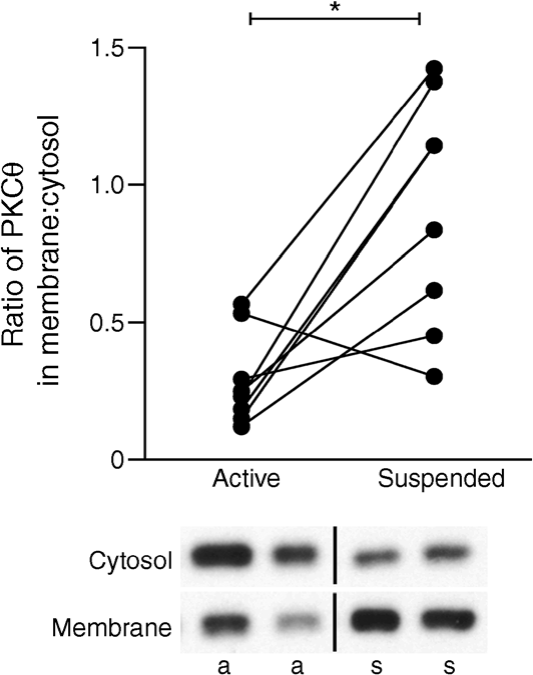
Ratio of PKCθ in the muscle membrane:cytosol (a measure of PKCθ activation) in the overnight fasted state. Representative blots are also shown (a, active; s, suspended). *n* = 8; *p < 0.05

It has been shown that activation of PKCθ by lipid intermediates, leading to translocation of PKCθ from the cytosol to the muscle membrane, is pivotal in lipid-induced insulin resistance (reviewed in [45]). Although human studies are limited in the amount of muscle material that can be obtained, which prevented us from performing lipidomics, we did determine PKCθ activation. We found that PKCθ translocation, reflected as the ratio of membrane/cytosol PKCθ content, was significantly higher in the suspended leg compared with the active leg (0.91 ± 0.16 vs 0.29 ± 0.06; *p* = 0.01; Fig. 5 [*n* = 8]).

**Fig. 6:**
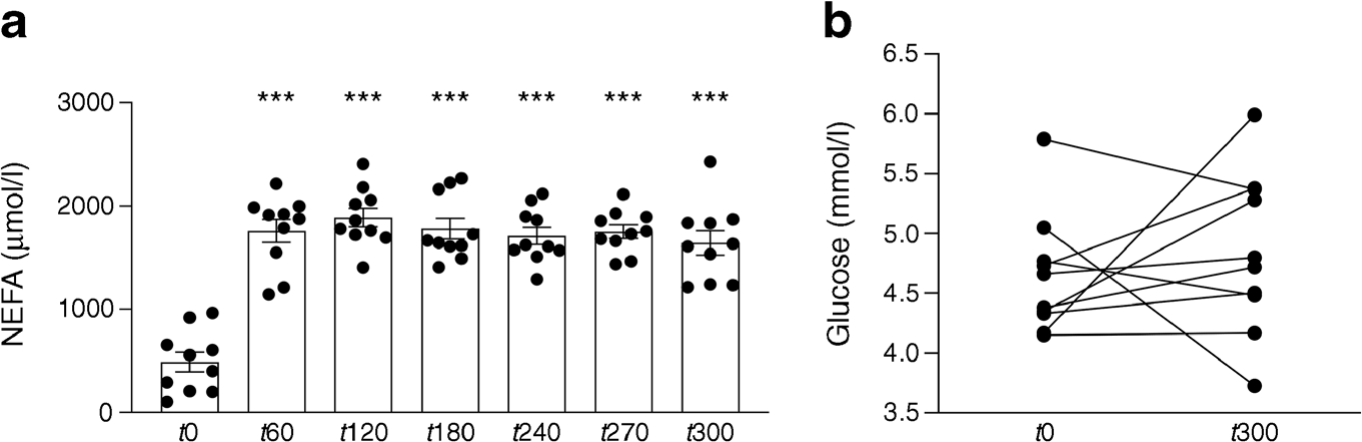
Plasma concentrations of (**a**) NEFA (*n* = 10; data expressed as mean ± SEM) and (**b**) glucose during the insulin+lipid infusion (*n* = 10). ***p < 0.001, compared with baseline (time [*t*]0)

To investigate if compromised mitochondrial oxidative capacity contributed to lipid-induced insulin resistance, we applied a model of lipid infusion. As expected, systemic plasma NEFA concentrations increased from 492 ± 95 μmol/l at baseline to 1759 ± 111 μmol/l 1 h after the start of lipid infusion and remained elevated over the 5 h lipid-infusion period (*p* < 0.0001; Fig. 6a). As aimed, plasma glucose concentrations were stable and did not change significantly after lipid and insulin infusion (4.6 ± 0.2 mmol/l at baseline and 4.8 ± 0.2 mmol/l after 5 h of lipid infusion; *p* = 0.462; Fig. 6b).

**Fig. 7:**
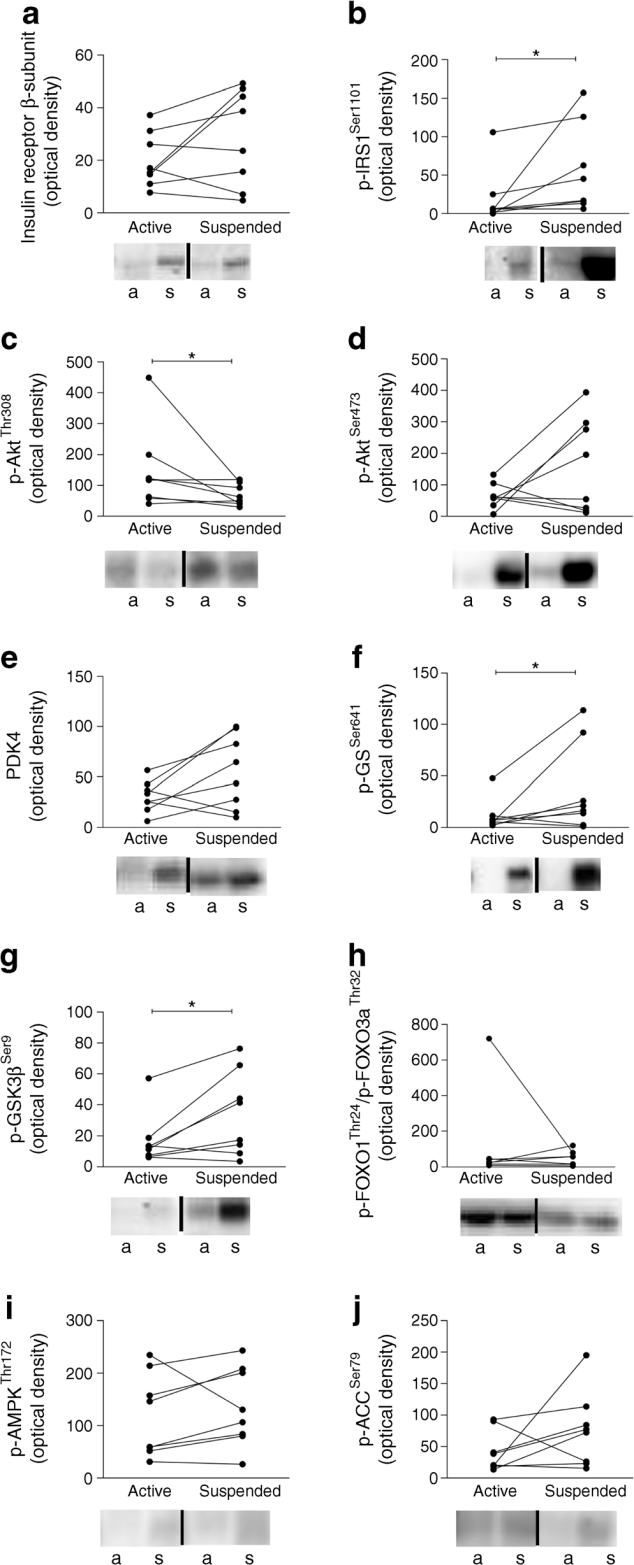
The expression level of (**a**) insulin receptor, (**b**) p-IRS1^Ser1101^, (**c**) p-Akt^Thr308^, (**d**) p-Akt^Ser473^, (**e**) PDK4, (**f**) p-GS^Ser641^, (**g**) p-GSK3β^Ser9^, (**h**) p-FOXO (FOXO1^Thr24^/FOXO3a^Thr32^), (**i**) p-AMPK^Thr172^ and (**j**) p-ACC^Ser79^ in the active vs the suspended leg, in the insulin-stimulated state. Representative blots also shown (a, active; s, suspended). *n* = 8; *p < 0.05

In contrast to rodent models, where ^14^C-labelled glucose can be used to determine leg-specific muscle glucose uptake, radioactive ^14^C cannot be applied in humans. Therefore, we measured insulin-induced activation of insulin signalling as a measure of insulin sensitivity in both the suspended and active leg upon a 5 h lipid infusion. As anticipated the expression level of insulin receptor between the suspended and the active leg after insulin+lipid infusion was unaffected (*p* = 0.161; Fig. 7a). Consistent with increased PKCθ activation in the suspended leg, we observed increased phosphorylation of IRS1 at Ser1101 which is a known target of PKCθ [46], in the suspended leg compared with the active leg (*p* = 0.018; Fig. 7b). Increased Ser1101 phosphorylation of IRS1, as well as attenuated phosphorylation of Akt at Thr308 and Ser473 residues, are expected under insulin-resistant conditions [12, 47]. Indeed, phosphorylation of Akt at Thr308 (but not Ser473) was significantly reduced in the suspended leg compared with the inactive leg upon insulin+lipid infusion (p-Akt^Thr308^: *p* = 0.036, Fig. 7c; p-Akt^Ser473^: *p* = 0.263; Fig. 7d). The abundance of PDK4 was borderline higher in the suspended leg upon insulin+lipid infusion compared with the active leg (*p* = 0.058; Fig. 7e). Furthermore, a higher phosphorylation of GS in the suspended leg compared with the active leg was observed (*p* = 0.050; Fig. 7f) and the phosphorylation of GSK3β at Ser9, inhibiting GSK3β activity, was significantly higher in the suspended leg compared with the active leg upon lipid infusion (*p* = 0.036; Fig. 7g). Finally, the phosphorylation of FOXO (*p* = 0.889; Fig. 7h), AMPK at Thr172 (*p* = 0.208; Fig. 7i) and ACC (*p* = 0.183; Fig. 7j) were not significantly different between legs. Collectively, these data indicate that the suspended leg featured more vigorous lipid-induced insulin resistance compared with the contralateral active leg.

## Discussion

Physical inactivity-associated low skeletal muscle mitochondrial oxidative capacity is suggested to contribute to skeletal muscle fat accumulation and insulin resistance [19, 20] and thereby is thought to be involved in the development of type 2 diabetes. However, proof for a direct relationship between these variables is difficult to examine in humans, since physical inactivity has many systemic effects that may also contribute to insulin resistance. Here we employed a unique human model of unilateral leg suspension that allowed us to investigate the direct effects of inactivity-mediated lowering of mitochondrial oxidative capacity on IMCL content and lipid-induced insulin resistance, using the contralateral leg as an active control. We observed that local physical inactivity indeed leads to lower in vivo mitochondrial oxidative capacity, consistent with previous findings [24], as well as a reduced fat oxidative capacity ex vivo, paralleled by elevated IMCL content, PKCθ activation and a blunted insulin action upon lipid infusion. Together these data demonstrate that the direct effects of physical inactivity on key metabolic parameters, including lowered mitochondrial oxidative capacity in muscle, may underlie lipid-induced insulin resistance, via the activation of PKCθ.

To investigate the local effects of physical inactivity independently from the systemic effects, we used the ULLS model in humans. During the ULLS period, leg skin temperature and overall activity level was lower in the suspended leg compared with the active leg, showing good compliance to the intervention. Importantly, however, the immobilised leg was not completely inactivated, which could underlie differences seen with studies including bed rest or a full leg cast, with more pronounced physical inactivity [48, 49]. Importantly, in our hands, this model of inactivity did not induce muscle mass loss, as substantiated by the lack of changes in leg muscle volume or muscle strength, likely due to the relatively short duration and relative mild induction of physical inactivity. This lack of loss of muscle mass is helpful in the present study as muscle mass is a determinant of insulin sensitivity. Together, the ULLS model provides the characteristics of a good model to mimic the modest reductions in physical activity in society that are associated with the development of chronic metabolic diseases over time.

Interestingly, both mitochondrial oxidative capacity and skeletal muscle fat oxidative capacity (palmitate oxidation to ^14^CO_2_) appear to be ~22% lower in the suspended leg compared with the active leg. Previously, we have shown that young, lean insulin-resistant offspring of parents with type 2 diabetes [11] and individuals with diabetes have a 25–48% lower mitochondrial oxidative capacity [21, 22, 50] and fat oxidative capacity [51] compared with healthy control participants, which was associated with increased IMCL. In addition, ageing has been shown to be associated with a 35–40% reduction in mitochondrial skeletal muscle oxidative/phosphorylation activity, increased IMCL content and muscle insulin resistance [52]. This indicates that with the relatively mild inactivity induced by ULLS, we created a reduction in mitochondrial oxidative capacity and fat oxidative capacity comparable with individuals prone to type 2 diabetes or diagnosed with type 2 diabetes.

The aim of the current study was to test the hypothesis that an inactivity-induced reduction in mitochondrial function would result in the accumulation of IMCL in humans and impaired insulin signalling upon lipid infusion. Indeed, we observed that IMCL content was higher in the suspended inactive leg than in the active leg upon the intervention period, as measured by two independent measures. This result is consistent with previous bedrest studies, showing a reduction in fat oxidation [53, 54], favouring incorporation of dietary saturated fatty acids (palmitate) into IMCLs [55]. In addition, our results are consistent with a recent study showing that 2 weeks of immobilisation also resulted in IMCL accumulation in young and elderly volunteers [56]. Although less likely, our model cannot rule out that physical inactivity resulted in a primary change in IMCL and secondary impairment in mitochondrial oxidative capacity. To investigate this, we used an ex vivo approach and determined the incorporation of [^14^C]palmitate into TAGs in muscle homogenates of the active and inactive leg. Consistent with our hypothesis, the reduced mitochondrial and fat oxidative capacity in muscle of the inactive leg was accompanied by higher [^14^C]palmitate incorporation into TAGs, suggesting that a low oxidative capacity accelerates IMCL accumulation, although this measure did not reach statistical significance probably due to the low number of participants in our study.

It has been shown that the association between IMCL accumulation and insulin resistance can be mechanistically explained by the interference of lipid intermediates with insulin signalling. In this respect, activation of novel PKCs has been shown to link fat accumulation with impaired insulin signalling in both animal models and individuals with type 2 diabetes [7]. Thus, studies using rats fed a high-fat diet showed that the translocation of PKC was associated with skeletal muscle insulin resistance [57]. In human studies, PKCθ translocation in the basal state was found to be higher in skeletal muscle of individuals with type 2 diabetes compared with lean control participants [12]. Here, we observed a pronounced increase in membrane-associated PKCθ content in the skeletal muscle of the inactivated compared with active leg, further providing evidence for the cascade by which a reduction in mitochondrial function leads to the accumulation of IMCLs and activation of PKCθ. We hypothesised that muscle of the inactive leg would be more insulin resistant upon lipid infusion compared with the active leg. Indeed, upon lipid infusion we found significantly higher p-GS^Ser641^, p-IRS1^Ser1101^ and lower p-Akt^Thr308^ in the suspended leg, all indicative of a more pronounced compromised insulin action in the suspended leg than in the active leg. Phosphorylation of IRS1 at Ser1101 inhibits Akt [12, 47], leading to reduced cellular glucose uptake. Phosphorylation of GS inactivates GS and results in lower glycogen production, which is suggestive of lower insulin sensitivity in the suspended leg. Also, an increase in PDK4 (which was non-significantly elevated in the suspended leg in our study) would be consistent with reduced insulin sensitivity in the inactive leg. Overall these data support the notion that the suspended, inactive leg, with compromised mitochondrial function, is more insulin resistant than the active leg upon lipid challenge. These data are consistent with our previous observation that highly trained athletes are protected against lipid-induced insulin resistance [43] and collectively suggest that a low mitochondrial function induced by physical inactivity predisposes humans to the development of lipid-induced insulin resistance. However, our results do not imply that mitochondrial function is the only determinant of insulin resistance; studies have shown that improvements in insulin sensitivity do not always coincide with improvements in mitochondrial function, for example, as was recently observed after gastric bypass-induced weight loss [58].

The intensive design of our study, including one-leg immobilisation for 12 consecutive days and two muscle biopsies, did prevent us from adding a control arm with the infusion of saline to compare difference in insulin sensitivity between legs without lipid infusion. Although this is a limitation of the study, the present design did allow us to carefully evaluate the differences between the active vs inactive leg under lipid-infused conditions, an intervention well-known to acutely induce insulin resistance of the periphery.

In conclusion, inactivity compromises in vivo mitochondrial oxidative capacity, along with reduced fat oxidative capacity and higher IMCL content, PKCθ translocation and an augmented decline in insulin action upon lipid infusion. Together, these data demonstrate the impact of physical inactivity on skeletal muscle insulin signalling, fat oxidation and IMCL storage and suggest that a reduced mitochondrial oxidative capacity may underlie these effects in healthy young men.

## Electronic supplementary material

ESM (PDF 1763 kb)

## Data Availability

The datasets generated and/or analysed during the current study are available from the corresponding author on reasonable request.

## Abbreviations

ACC: Acetyl-CoA carboxylase
AMPK: AMP-activated protein kinase
ASM: Acid-soluble metabolite
CAM: CIRO Activity Monitor
DAG: Diacylglycerol
FOXO: Forkhead box O
GS: Glycogen synthase
GSK3β: Glycogen synthase kinase 3β
^1^H-MRS: Proton magnetic resonance spectroscopy
IMCL: Intramyocellular lipid
NSA: Number of signal averages
PCr: Phosphocreatine
PDK4: Pyruvate dehydrogenase kinase 4
PKC: Protein kinase C
^31^P-MRS: Phosphorous magnetic resonance spectroscopy
T_1/2_: Half-time
TAG: Triacylglycerol
TE: Echo time
TR: Repetition time
TSE: Turbo spin echo
ULLS: Unilateral lower-limb suspension
