# Supplementary material for: One-leg inactivity induces a reduction in mitochondrial oxidative capacity, intramyocellular lipid accumulation and reduced insulin signalling upon lipid infusion: a human study with unilateral limb suspension
Source: Diabetologia. 2020 Mar 17;63(6):1211–22. doi: 10.1007/s00125-020-05128-1 (PMC7228997; doi:10.1007/s00125-020-05128-1)
Supplement: Supplementary file 1 — (PDF 1763 kb) [file 125_2020_5128_MOESM1_ESM.pdf]

ESM table 1

ESM Table 1

Name	Epitope	Size (kDa)	Source	Company	Reference	
pyruvate dehydrogenase kinase 4 (PDK4)	C terminal region of Human PDK4	48	Rabbit	Abcam	# ab89295	https://www.abcam.com/pdk4-antibody-ab89295.html
Insulin Receptor b-subunit	C terminal 19 amino acids of the human inulin receptor beta-subunit	95	Rabbit	Santa Cruz Biotechnology	sc-711	https://www.scbt.com/p/insulin-rbeta-antibody-c-19
pIRS1^{Ser1101}	phospho-Ser1101	180	Rabbit	Cell Signaling Technology	#2385	https://www.cellsignal.com/products/primary-antibodies/phospho-irs-1-ser1101-antibody/2385
pAkt^{Thr308}	phospho-Thr308	60	Rabbit	Cell Signaling Technology	#9275	https://www.cellsignal.com/products/primary-antibodies/phospho-akt-thr308-antibody/9275?site-search-type=Products&N=4294956287&Ntt=%239275&fromPage=plp&_requestid=218704
pAkt^{Ser473}	phospho-Ser473	60	Rabbit	Cell Signaling Technology	#9271	https://www.cellsignal.com/products/primary-antibodies/phospho-akt-ser473-antibody/9271?site-search-type=Products&N=4294956287&Ntt=%239271&fromPage=plp&_requestid=218740
phosphorylated glycogen synthase kinase 3β (pGSK3β^{Ser9})	phospho-Ser9	46	Rabbit	Cell Signaling Technology	#9323	https://www.cellsignal.com/products/primary-antibodies/phospho-gsk-3b-ser9-5b3-rabbit-mab/9323?site-search-type=Products&N=4294956287&Ntt=%23+9323&fromPage=plp&_requestid=218774
phosphorylated glycogen synthase (pGS^{Ser641})	phospho-Ser641	85	Rabbit	Cell Signaling Technology	#3891	https://www.cellsignal.com/products/primary-antibodies/phospho-glycogen-synthase-ser641-antibody/3891
phosphorylated Forkhead box O (pFOXO1^{Thr24}/FOXO3a^{Thr32})	phospho-FoxO1-Thr24 / phospho-FoxO3a-Thr32	80, 95	Rabbit	Cell Signaling Technology	#9464	https://www.cellsignal.com/products/primary-antibodies/phospho-foxo1-thr24-foxo3a-thr32-antibody/9464
phosphorylated AMP-activated protein kinase (pAMPK^{Thr172})	phospho-Thr172	62	Rabbit	Cell Signaling Technology	#2535	https://www.cellsignal.com/products/primary-antibodies/phospho-ampka-thr172-40h9-rabbit-mab/2535?site-search-type=Products&N=4294956287&Ntt=%232535&fromPage=plp&_requestid=219025
phosphorylated acetyl-CoA carboxylase (pACC^{Ser79})	phospho-Ser79	280	Rabbit	Cell Signaling Technology	#3661	https://www.cellsignal.com/products/primary-antibodies/phospho-acetyl-coa-carboxylase-ser79-antibody/3661?site-search-type=Products&N=4294956287&Ntt=%233661&fromPage=plp&_requestid=219053
**Anti-Rabbit IgG (H+L), HRP Conjugate**	anti-rabbit IgG		Goat	Promega	W4011	https://www.promega.de/products/imaging-and-immunological-detection/elisas-and-antibodies/anti_rabbit-igg-h-and-l-hrp-conjugate/?catNum=W4011

all antibodies and bufers were used according to the supplier's instructions (see hyperlink)

dilution primary antibody 1:1000

dilution primary antibody 1:2500

ESM figure 1

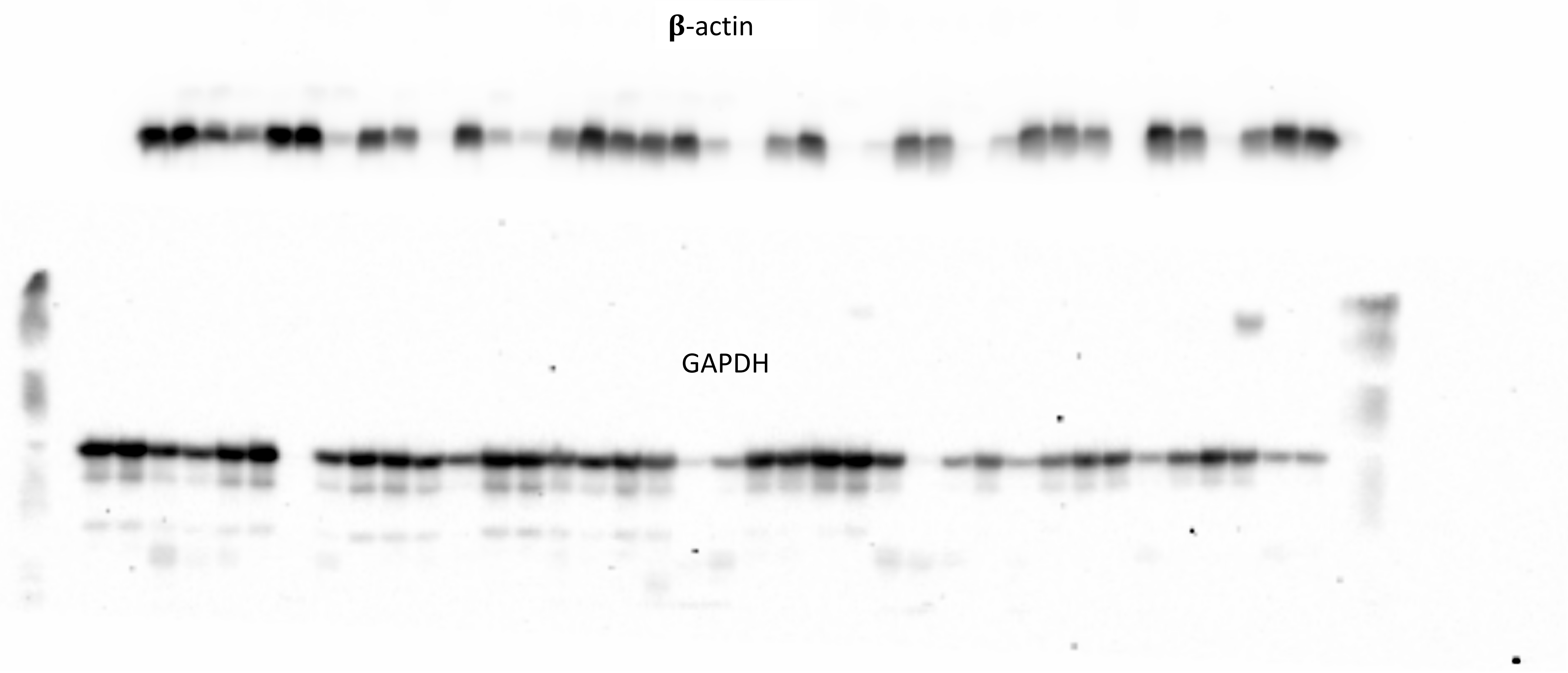

Western blots were normalized for the abundance of β-actin and GAPDH, shown in this figure.
